# Anti‐HPA‐1a IgG3 subclass antibodies induce strong platelet phagocytosis

**DOI:** 10.1111/bjh.70533

**Published:** 2026-05-14

**Authors:** Silke Schmidt, Xiuzhang Xu, Gabriela Michel, Hartmut Kroll, Gregor Bein, Sentot Santoso

**Affiliations:** ^1^ Institute for Clinical Immunology, Transfusion Medicine and Hemostasis Justus‐Liebig‐University Giessen Giessen Germany; ^2^ Institute of Blood Transfusion and Hematology, Guangzhou Blood Centre Guangzhou Medical University Guangzhou China; ^3^ The Key Medical Laboratory Guangzhou China; ^4^ Institute for Transfusion Medicine Dessau Red Cross Blood Transfusion Service NSTOB Dessau Germany

**Keywords:** anti‐HPA‐1a, FNAIT, IgG‐subclass, prediction, severity

To the Editor,

Fetal and neonatal alloimmune thrombocytopenia (FNAIT) is a serious bleeding condition caused by maternal Immunoglobulin G (IgG) alloantibodies reacting with platelet antigens expressed on fetal platelets. In Caucasian populations, anti‐human platelet antigen‐1a (anti‐HPA‐1a) alloantibodies are responsible for about 85% of all FNAIT cases. The clinical presentation of FNAIT varies from asymptomatic thrombocytopenia to severe clinical complications, such as intracranial haemorrhage. However, laboratory testing as a prognostic tool to identify fetuses/newborns at risk of severe FNAIT is still lacking.^1^

Recently, the international consensus study on FNAIT (Delphi) agreed that anti‐HPA‐1a antibody levels could be used to identify pregnancies with increased degree of fetal thrombocytopenia and bleeding risk. However, no consensus could be achieved for a clinically useful cut‐off value with adequate sensitivity and specificity.^2^ Apparently, clearance of anti‐HPA‐1a opsonized fetal platelets by monocytes/macrophages via Fc‐dependent pathway represents the major mechanism responsible for thrombocytopenia in FNAIT cases.^3^ Accordingly, our study showed that monocytes are responsible for the Fc‐dependent platelet phagocytosis via Fc gamma receptor I (FcγRI) and Fc gamma receptor IIIa (FcγRIIIa). Furthermore, we found high correlation between platelet phagocytosis rate measured by whole platelet phagocytosis assay (WHOPPA) and anti‐HPA‐1a antibody levels.^4^

Human IgG antibodies comprise IgG1, IgG2, IgG3 and IgG4 subclasses and each IgG subclass has distinct binding affinity for different FcγRs leading to variations in antibody‐dependent cellular phagocytosis (ADCP).^5^ Previous studies showed that IgG1 and IgG3 subclasses were frequently detected in FNAIT (96%) caused by anti‐HPA‐1a antibodies and the levels of IgG3 antibody subclass were significantly higher in the severe compared to mild cases.^6^, ^7^ Contrary, another study showed that neither antibody titre nor IgG subclass alone could predict the severity or the occurrence of thrombocytopenia in newborns.^8^ This discrepancy could be attributed to different parameters including cohort size, time of sampling and method used for IgG subclass identification.

In 2015, Eksteen and colleagues succeeded to generate a human platelet antigen‐1a‐specific monoclonal antibody (mAb) derived from a B cell from a woman alloimmunized in pregnancy (named 26.4; IgG3).^9^ Based on the known sequence of this mAb, we produced recombinant mAb 26.4 IgG subclasses (Table S1) to compare their capability to induce platelet phagocytosis by WHOPPA.

To validate the IgG subclass specificity, we incubated platelets with mAb 26.4 IgG1, IgG2, IgG3, IgG4 and IgG1 LALAPG containing L234A, L235A and P329G mutations in the Fc region leading to elimination of immune effector function (Fc‐silencing). Subsequently, platelets were labelled with Phycoerythrin (PE)‐conjugated mAb specific for IgG subclasses and analysed by flow cytometry using AF488‐labelled anti‐human Fc as positive control. Platelets were identified by forward scatter (FSC) and side scatter (SSC) and by staining with mAbs against CD41 and CD61 (Figure S1). When platelets were opsonized with mAb 26.4 IgG1, a positive reaction was only detected with anti‐IgG1, but not with anti‐IgG2, ‐IgG3 and IgG4 (Figure 1A). Similar results were obtained with mAb 26.4 IgG2, IgG3, IgG4 and IgG1 LALAPG when tested with their corresponding anti‐IgG subclass. Negative controls with human IgG (hIgG) also showed no reaction. Strikingly, compared to other IgG subclasses, incubation of platelets with mAb 26.4 IgG2 subclass resulted in broader fluorogram (Figure 1A, arrow) and elongated FSC/SSC dot plot (Figure S2). This phenomenon was observed with all four anti‐IgG subclasses indicating that mAb 26.4 IgG2 subclass induced the formation of platelet aggregates.

**FIGURE 1:**
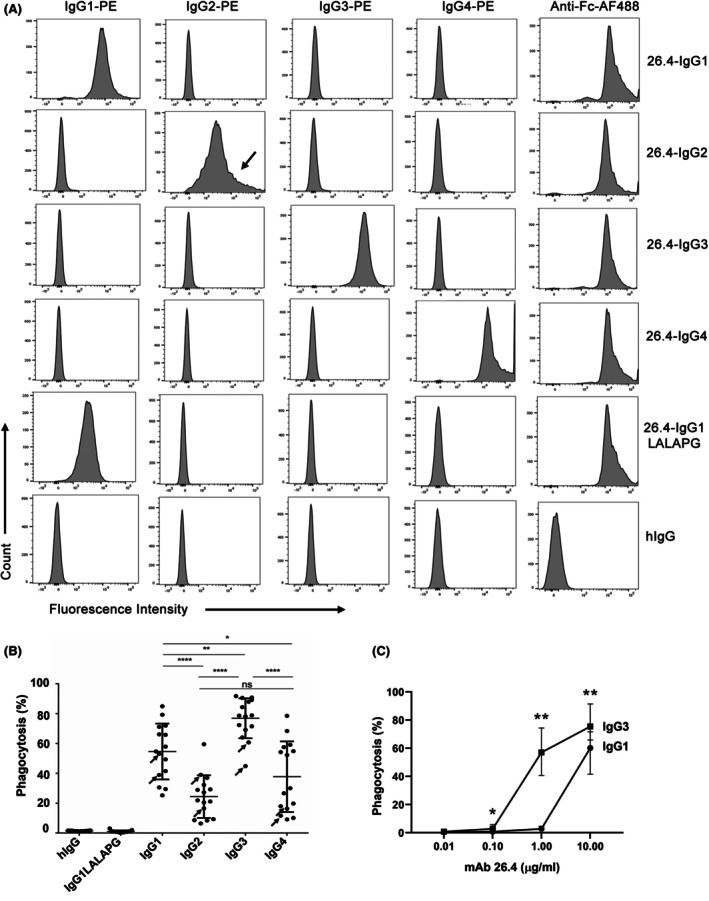
(A) Analysis of platelets bound monoclonal antibody (mAb) 26.4 IgG subclasses by flow cytometry. Washed platelets were incubated with mAb 26.4 IgG1, IgG2, IgG3, IgG4 or IgG1 LALAPG (2.5 μg/mL) or normal human IgG (hIgG; 0.4 μg/mL). After washings, platelets were stained with PE‐labelled anti‐human IgG1, IgG2, IgG3 or IgG4 subclass (1 μg/mL) as indicated. A488‐conjugated antibody against human Fc was run as positive control. Antibody‐stained platelet population (see Figure S1A,B) were analysed by median fluorescence intensity (MFI). (B) Analysis of platelet phagocytosis caused by mAb 26.4 different IgG subclasses. PhRodo‐labelled HPA‐1aa homozygous platelets (*n* = 15) were opsonized with mAb 26.4 IgG1, IgG2, IgG3 or IgG4 subclass (10 μg/mL) using human IgG and effector silencing mAb 26.4 IgG1 LALAPG (10 μg/mL) as negative controls. Opsonized platelets were then incubated with platelet‐free whole blood and the percentage of pHrodo^+^, CD14^+^ monocytes (% phagocytosis) that engulfed platelets were measured by flow cytometry (Table S2). Platelet phagocytosis by monocytes from two FF_158_ FcγRIIIa homozygous individuals (D68 and D81; Table S2) are indicated by arrows. Bars indicate means ± SEM. Statistical analysis was performed with one‐way ANOVA with Bonferroni's correction for multiple comparisons. ****p < 0.0001, **p < 0.01, *p < 0.05; n.s: Not significant. (C) Analysis of platelet phagocytosis caused by mAb 26.4 IgG1 and IgG3 subclasses at different concentrations. PhRodo‐labelled platelets were stained with mAb 26.4 IgG1 or IgG3 subclass (0.01–10.00 μg/mL) as indicated. The percentage of monocytes that engulfed platelets was measured by flow cytometry as above.

Afterwards, we compared the clearance of pHrodo‐labelled HPA‐1aa platelets from different donors (*n* = 15) opsonized with anti‐HPA‐1a subclasses (10 μg/mL) by WHOPPA. The rates of monocytes (CD14^+^, pHrodo^+^) that engulfed platelets were measured. As shown in Figure 1B, IgG3 caused the highest platelet phagocytosis rates followed by IgG1, IgG4 and IgG2. As expected, hIgG and mAb 26.4 IgG1 LALAPG did not induce platelet phagocytosis. Furthermore, antibody titration showed that IgG3 was 10 times more efficient in triggering platelet phagocytosis compared to IgG1 antibody subclass (1 vs. 10 μg/mL, Figure 1C).

In accordance with our previous finding,^4^ all IgG antibody subclasses that interact with FcγRI and FcγRIIIa caused high platelet phagocytosis in the following order: IgG3 > IgG1 > IgG4 > IgG2. The weakly binding IgG2 antibody subclass could only trigger low platelet phagocytosis.^5^ Previous study showed that an IgG2 subclass of anti‐D mAb also exhibited low phagocytic activity.^10^

Although IgG1, IgG3 and IgG4 subclasses bound to the high affinity FcγRI with comparable affinity constants (650, 610, 340 × 10^5^ M^−1^, respectively),^11^, ^12^ IgG3 antibody induced significantly higher phagocytosis rates compared to the other two subclasses. The IgG3 subclass has unique physiochemical characteristics due to its extended hinge region leading to increased flexibility. This allows IgG3 to interact more effectively with target antigens and its ability to mediate effector functions. Consequently, IgG3 antibody bound to FcγRIIIa with significant higher affinity (around 40–140 times) compared to other IgG subclasses.^13^ In humans, two allotype variants of FcγRIIIa (V158 and F158) exist. In comparison to the F158 alloform, V158 interacts with IgG3 with higher affinity (98 vs. 77 × 10^−5^ M).^5^, ^11^ Genotyping of V158 and F158 variant by Taqman showed that mAb 26.4 IgG3 caused low phagocytosis rated in two donors, typed as FF homozygous (Figure 1B; Table S2), whereas the remaining 13 donors with higher phagocytosis rates were heterozygous F158V. These results further support the unique role of anti‐HPA‐1a IgG3 subclass as a potent trigger for platelet phagocytosis.

However, a previous study found that the phagocytosis rate of 26.4 IgG3 opsonized platelets did not significantly differ from mAb 26.4 IgG1.^4^ This discrepancy most probably results from a different method used by these authors. They used green CMFDA (Cell‐Permeable Cell Tracer) labelled platelets, which did not allow direct discrimination between engulfed platelets and platelets that adhere to the monocyte cell surface. In our study, we used pHrodo‐labelled platelets allowing detection of engulfed platelets only by ‘untouched’ monocytes in blood.

Although mAb 26.4 IgG2 did not induce significant platelet phagocytosis in WHOPPA, this antibody subclass apparently led to platelet activation. Recently, a similar phenomenon was observed with an IgG2 anti‐HLA‐I.^13^ However, it is currently unclear whether these activated platelets can be cleared via Fc‐independent pathway in the liver.^14^ Even less is known about the role of IgG4 in platelet phagocytosis. Previous study indicated a potential of human IgG4 to inhibit FcγR activation pathways.^15^ Our results, however, showed that anti‐HPA‐1a IgG4 subclass could induce strong platelet phagocytosis in some individuals (about half), indicating the role of certain FcγR dimorphism (Figure 2). This, however, did not relate to H131R dimorphism in FcγRIIa (*data not shown*).

**FIGURE 2:**
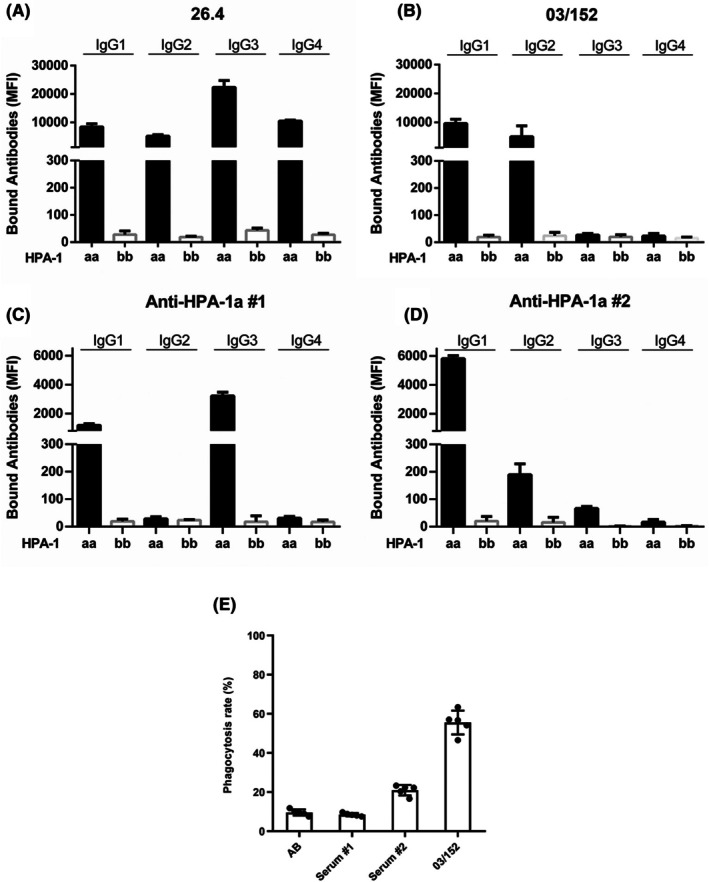
Analysis of anti‐HPA‐1a antibodies by Luminex. Binding monoclonal antibody (mAbs) 26.4 IgG1, IgG2, IgG3 and IgG4 subclasses (A), standard anti‐HPA‐1a serum (B) and two anti‐HPA‐1a sera (#1 and #2) (C, D) were analysed by Luminex bead‐based platelet antibody detection method (PAKLx). HPA‐1aa or HPA‐1bb beads were incubated with 10 μL mAb 26.4 IgG1, IgG2, IgG3 or IgG4 subclass (10 μg/mL), 10 μL anti‐HPA‐1a standard serum (undiluted; 100 IU) or anti‐HPA‐1a serum samples (undiluted). After washings, bound antibodies were labelled with 50 μL PE‐fluorescence‐labelled anti‐human IgG1, IgG2, IgG3 or IgG4 subclass (1 μg/mL). Antibody bound onto the beads was then analysed by Luminex 200 fluoroanalyser using Lifecodes Matchit Platelet Antibody software. Bars represent mean ± SEM of triplicates from three independent experiments. (E) Analysis of platelet phagocytosis by whole platelet phagocytosis assay (WHOPPA). PhRodo‐labelled HPA‐1aa homozygous. Platelets (*n* = 5) were opsonized with normal AB serum, serum #1, serum #2 and 03/152 reference serum. The percentage of monocytes that engulfed platelets were measured as above. Bars indicate means ± SEM.

Currently, antigen capture assays (such as MAIPA) represent the standard method for platelet antibody detection. However, accumulated studies performed by MAIPA showed low positive predictive values (54%–97%) leading to the general view that the determination of maternal antibody level by MAIPA is unsuitable guiding antenatal treatment (see Supporting Information: reference 16). Since this enzyme‐based approach is highly depending on pH and temperature, it needs therefore careful control of reaction conditions and correct antibody standard, particularly for quantitative analyses. Accordingly, our recent study showed a stronger correlation between phagocytosis rate and bound anti‐HPA‐1a when antibody binding was measured by flow cytometry rather than by MAIPA.^4^

Meanwhile, Luminex bead‐based platelet antibody detection method (PAKLx; Immucor) is proved to be a simple and reliable method to detect anti‐HPA‐1a antibodies in serum (see Supporting Information: reference 17). Therefore, we asked the question whether PAKLx represents a reliable method to differentiate anti‐HPA‐1a antibody subclasses. HPA‐1aa and HPA‐1bb beads were incubated with mAb 26.4 IgG1, IgG2, IgG3 or IgG4, stained with PE‐labelled anti‐human IgG subclasses and analysed by Luminex 200 fluoroanalyser using Lifecodes Matchit Platelet Antibody software (Immucor). No reaction with HPA‐1bb beads was obtained. Contrary, strong reactions were detected with HPA‐1aa beads (Figure 2A). Notably, IgG3 antibody subclass showed a higher binding rate compared to other IgG subclasses (MFI around 20.000 vs. 10.000). This is probably due to the novel hinge‐folding mode flexibility between Fab arms.^11^ In the control experiment, cross‐reactivity was not found between anti‐human IgG subclasses (data not shown).

We finally analysed the distribution of IgG subclasses in the anti‐HPA‐1a reference sera (03/152; NIBSC) consisting of a pooled plasma from six donors and tested randomly two anti‐HPA‐1a sera (serum #1 and serum #2) from FNAIT cases. Surprisingly, 03/152, the only standard serum available worldwide, only contained IgG1 and IgG2, but not IgG3 and IgG4 subclasses (Figure 2B). In contrast, serum #1 contained IgG3 and IgG1 and serum #2 IgG1, IgG2 and IgG3 (Figure 2C,D). Although IgG3 antibody subclass was detectable in these samples, analysis by WHOPPA showed low phagocytosis activity (Figure 2E) indicating that apart from the IgG subclass, the antibody titre plays a critical role.

Furthermore, Kapur and colleagues showed that decreased levels of Fc fucosylation of anti‐HPA‐1a‐specific IgG1 correlated with FNAIT severity (see Supporting Information: reference 17). Therefore, the impact of IgG3‐Fc fucosylation of anti‐HPA‐1a should also be considered and investigated further.

All in all, it remains to be seen whether IgG subclass‐specific anti‐HPA‐1a testing offers more accurate approach to risk stratification. This will require a new anti‐HPA‐1a standard including representative quantities of all IgG subclasses replacing 03/152. Over and above, a larger number of FNAIT cases is mandatory to dissect which of these factors, titre, subclass and fucosylation are critical for predicting disease severity.

## AUTHOR CONTRIBUTIONS

SiS, XX and GM performed the experiments and analysed the data. GB and SeS designed research, analysed the data and wrote the manuscript. HR provided important reagents and critical prereview.

## CONFLICT OF INTEREST STATEMENT

The authors have no conflict of interest.

## Supporting information


Data S1.

## Data Availability

The data that support the findings of this study are available from the corresponding author upon reasonable request.
