# Supplementary material for: Anti‐HPA‐1a IgG3 subclass antibodies induce strong platelet phagocytosis
Source: Br J Haematol. 2026 May 14;209(1):358–62. doi: 10.1111/bjh.70533 (PMC13340540; doi:10.1111/bjh.70533)
Supplement: Supplementary file 1 — Data S1. [file BJH-209-358-s001.zip › ANTI-HPA-1a (Manuscript-R3.3.2 clean).docx]

**SUPPLEMENTARY**

**MATERIALS AND METHODS**

***Antibodies***

Recombinant human anti-HPA-1a mAb (clone 26.4) IgG1, IgG2, IgG3, IgG4 subclasses and effector silencing anti-HPA-1a IgG1 LALAPG were produced based on the published IgG3 sequence of mAb 26.4 (Eksteen et al., 2025; European Patent Specification WO 2015/150417) using antibody subclass switching approach in Chinese Hamster Ovary (CHO) cells (Biointron, Shanghai, China). Antibody concentration was determined by Nanodrop and the purity (>95%) of the antibody was analyzed by SDS-PAGE under reducing conditions. Phycoerythrin (PE)-labelled anti-human IgG1 Hinge (clone 4E3), IgG2 Fc (clone 31-7-4), IgG3 Hinge (clone HP6050), IgG4 Fc (clone HP6025) were purchased from Southern Biotech (Biozol, Eching, Germany). All PE-conjugated was supplied as 0.1 mg in 1.0 ml PBS/NaN_3_ with the same Degree of Labeling (DOL) of 1.0-1.5. Normal human IgG was from Sigma Aldrich (Darmstadt, Germany). Alexa Fluor-488 (AF-488) conjugated rabbit-anti-human against Fcγ was from Dianova (Hamburg, Germany). Allophycocyanin **(**APC)-labelled mAb against CD41 and Fluorescein isothiocyanate (FITC)-labelled mAb against CD61 were from Biolegend (San Diego, CA, USA) Anti-HPA-1a standard (03/152; 100 IU), a pooled human plasma derived from six immunized donors against HPA-1a were from the NIBSC (Potters Bar Hertfordshire, UK). Currently, 03/152 is the only reference standard for anti-HPA-1a available worldwide. Anti-HPA-1a sera (#1 and #2) were from FNAIT cases. This study was conducted in accordance with the declaration of Helsinki and was approved by the Ethics Committee of the Medical Faculty, Justus Liebig University, Giessen, Germany (files no. 82/09 and 05/00). Patient consent was waived due to identified analysis of retrospective data or blood samples.

***Platelet antibody testing by flow cytometry***

Platelets were isolated from ACD anticoagulated blood from HPA-1a typed donors. After centrifugation (25 min, 110g), platelet-rich plasma (PRP) was isolated and washed twice (10 min, 800 g) with Dulbecco’s Phosphate-Buffer Saline (D-PBS; Anprotec, Bruckberg, Germany) containing 10 mM EDTA and 0.3 μM prostaglandin (Sigma-Aldrich, St. Louis, Missouri, USA) (PEP, pH7.4). Aliquots of 100 μl platelets (10^6^ platelets/ml PEP) were incubated with mAb 26.4 (2.5 μg/ml) for 30 min. Normal human IgG was run as negative control. Platelets were then washed with PEP and then stained with PE-conjugated mAbs against human IgG subclasses and with AF-488-labelled mAb against Fc (1 μg/ml) for 30 min in the dark. After washing, platelets were suspended in 200 μl PEP and analyzed by flow cytometry using BD FACS Canto II Flow Cytometer (BD Biosciences, San Jose, CA, USA). Platelets were identified according to their forward scatter (FSC) and side scatter (SSC) characteristics. In total, 10^4^ platelets were counted.

***Whole Blood Platelet Phagocytosis Assay (WHOPPA)***

Blood was collected from different HPA-1aa homozygous donors (n = 15). Phagocytosis of anti-HPA-1a-opsonized platelets by monocytes was performed by WHOPPA as previously described.^9^ In brief, aliquots of 250µl of pHrodo-labelled HPA-1aa homozygous platelets (5x10^7^ platelets) were opsonized with diluted recombinant anti-HPA-1a IgG subclass (10 μg/ml), 62.5 μl anti-HPA-1a standard (03/152; see above) or serum (undiluted) for 30 min in the dark. Opsonized platelets (1.5 x 10^7^ platelets) were incubated with platelet-free whole blood containing 5 x 10^4^ monocytes. The phagocytosis rate was defined as the percentage of monocytes that engulfed platelets (CD14^+^ and pHrodo^+^) by flow cytometry (see above). Platelets and monocytes from the same donor were used for each experiment. Experiments were performed in a group of 2-3 blood donors. In parallel, DNA was isolated from these blood donors (n =15) and genotyped for FcγRIIa and FcγRIIIa polymophisms (see below).

**Genotyping of FcγRIIa and FcγRIIIa by Taqman assay**

Genotyping of FcγRIIa (rs1801274; A/G substitution) and FcγRIIIa dimorphism (rs396991; C/A substitution) by TaqMan^TM^ Assay was performed as recommended by the manufacturer (Assay ID C_25815666_10; Thermo Fisher Scientific, Dreieich Germany).

***Typing of anti-HPA-1a antibody IgG subclasses by Luminex***

IgG subclasses of anti-HPA-1a antibodies were analyzed by Luminex bead-based assay (PAKLx) (Immucor, Dreieich, Germany). In brief, 40 μl of HPA-1aa and HPA-1bb beads were incubated with 10 μl mAb 26.4 IgG subclass (10 μg/ml), undiluted anti-HPA-1a sera for 60 min. After washings, aliquots of 5 μl PE-labelled mAbs against human IgG subclasses (in 45 μl dilution buffer (1:100; Immucor) were added for 30 min. Antibody bound onto the beads in 150 μl dilution buffer was analyzed by Luminex 200 Fluoroanalyzer (LABScan 3D, One Lambda) using Lifecodes Matchit Platelet Antibody software (Immucor).

***Statistical analysis***

Statistical analysis was performed with 1-way Anova with Bonferroni’s correction for multiple comparisons.

**REFERENCES**

16. Kjaer M, Bertrand G, Bakchoul T, et al. Maternal HPA-1a antibody level and its role in predicting the severity of Fetal/Neonatal Alloimmune Thrombocytopenia: a systematic review. Vox Sanguinis. 2019;114(1):79–94.

17. Cooper N, Bein G, Heidinger K, Santoso S, Sachs UJ. A bead-based assay in the work-up of suspected platelet alloimmunization. Transfusion. 2016;56(1):115–8.

18. Kapur R, Kustiawan I, Vestrheim A, et al. A prominent lack of IgG1-fucosylation of platelet alloantibodies in pregnancy. Blood 2014;123(4):471-80.

**LEGENDS FOR THE SUPPLEMENTARY FIGURES**

**Figure 1S: Characterization of platelet population by flow cytometry**
A) Platelet populations were analyzed by forward scatter (FSC) and (SSC) and B) by specific labelling with APC-labelled mAb against CD41 and FITC-conjugated mAb against CD61.
C represents isotype control.

**Figure 2S: Analysis of platelets bound mAb 26.4 IgG subclasses by flow cytometry**
Washed platelets were incubated with mAb 26.4 IgG1, IgG2, IgG3, IgG4 or IgG1 LALAPG (2.5 μg/ml) or human IgG (hIgG; 0.4 μg/ml). After washings, platelets were stained with PE-labelled anti-human IgG1, IgG2, IgG3 or IgG4 subclass (1 μg/ml) as indicated. AF488-conjugated mAb against Fc antibody (3.75 μg/ml) was run as positive control. Platelet populations were analyzed by forward scatter (FSC) and (SSC)

**Tables 1-1 and 1-2: Amino acid sequences of all recombinant anti-HPA-1a antibodies used in this study.**

**Table 2: Genotyping of V158F dimorphism of blood donors (n = 15) analyzed by Taqman.**
D: Number of the donors; %: Phagocytosis rates; Homozygous FF158 donors are underlined.
