# Supplementary material for: Anti‐HPA‐1a IgG3 subclass antibodies induce strong platelet phagocytosis
Source: Br J Haematol. 2026 May 14;209(1):358–62. doi: 10.1111/bjh.70533 (PMC13340540; doi:10.1111/bjh.70533)
Supplement: Supplementary file 1 — Data S1. [file BJH-209-358-s001.zip › ANTI-HPA-1a (Tables R3.3).docx]

**Table 1-1 Supplementary**

**F22474201 (26.4-IgG1)**

MHSSALLCCLVLLTGVRA

QVQLQQSGPGLVKPSQTLSLTCAISGDSVSSNSAAWNWIRQSPSRGLEWLGRTYFRSNWYNDYAASVKSRITINQDTSKNQLSLQLNSVTPEDTAMYYCARDGAWGGSSWWPGLPHHYYSGMDVWGQGTTVTVSS

ASTKGPSVFPLAPSSKSTSGGTAALGCLVKDYFPEPVTVSWNSGALTSGVHTFPAVLQSSGLYSLSSVVTVPSSSLGTQTYICNVNHKPSNTKVDKKVEPKSCDKTHTCPPCPAPELLGGPSVFLFPPKPKDTLMISRTPEVTCVVVDVSHEDPEVKFNWYVDGVEVHNAKTKPREEQYNSTYRVVSVLTVLHQDWLNGKEYKCKVSNKALPAPIEKTISKAKGQPREPQVYTLPPSRDELTKNQVSLTCLVKGFYPSDIAVEWESNGQPENNYKTTPPVLDSDGSFFLYSKLTVDKSRWQQGNVFSCSVMHEALHNHYTQKSLSLSPGK

**F22474202 (26.4-IgG2)**

MHSSALLCCLVLLTGVRA

QVQLQQSGPGLVKPSQTLSLTCAISGDSVSSNSAAWNWIRQSPSRGLEWLGRTYFRSNWYNDYAASVKSRITINQDTSKNQLSLQLNSVTPEDTAMYYCARDGAWGGSSWWPGLPHHYYSGMDVWGQGTTVTVSS

ASTKGPSVFPLAPCSRSTSESTAALGCLVKDYFPEPVTVSWNSGALTSGVHTFPAVLQSSGLYSLSSVVTVPSSNFGTQTYTCNVDHKPSNTKVDKTVERKCCVECPPCPAPPVAGPSVFLFPPKPKDTLMISRTPEVTCVVVDVSHEDPEVQFNWYVDGVEVHNAKTKPREEQFNSTFRVVSVLTVVHQDWLNGKEYKCKVSNKGLPAPIEKTISKTKGQPREPQVYTLPPSREEMTKNQVSLTCLVKGFYPSDIAVEWESNGQPENNYKTTPPMLDSDGSFFLYSKLTVDKSRWQQGNVFSCSVMHEALHNHYTQKSLSLSPGK

**B22680401 (26.4-IgG3)**

MHSSALLCCLVLLTGVRA

QVQLQQSGPGLVKPSQTLSLTCAISGDSVSSNSAAWNWIRQSPSRGLEWLGRTYFRSNWYNDYAASVKSRITINQDTSKNQLSLQLNSVTPEDTAMYYCARDGAWGGSSWWPGLPHHYYSGMDVWGQGTTVTVSS

ASTKGPSVFPLAPCSRSTSGGTAALGCLVKDYFPEPVTVSWNSGALTSGVHTFPAVLQSSGLYSLSSVVTVPSSSLGTQTYTCNVNHKPSNTKVDKRVELKTPLGDTTHTCPRCPEPKSCDTPPPCPRCPEPKSCDTPPPCPRCPEPKSCDTPPPCPRCPAPELLGGPSVFLFPPKPKDTLMISRTPEVTCVVVDVSHEDPEVQFKWYVDGVEVHNAKTKPREEQYNSTFRVVSVLTVLHQDWLNGKEYKCKVSNKALPAPIEKTISKTKGQPREPQVYTLPPSREEMTKNQVSLTCLVKGFYPSDIAVEWESSGQPENNYNTTPPMLDSDGSFFLYSKLTVDKSRWQQGNIFSCSVMHEALHNRFTQKSLSLSPGK

**B22680402 (26.4-IgG4)**

MHSSALLCCLVLLTGVRA

QVQLQQSGPGLVKPSQTLSLTCAISGDSVSSNSAAWNWIRQSPSRGLEWLGRTYFRSNWYNDYAASVKSRITINQDTSKNQLSLQLNSVTPEDTAMYYCARDGAWGGSSWWPGLPHHYYSGMDVWGQGTTVTVSS

ASTKGPSVFPLAPCSRSTSESTAALGCLVKDYFPEPVTVSWNSGALTSGVHTFPAVLQSSGLYSLSSVVTVPSSSLGTKTYTCNVDHKPSNTKVDKRVESKYGPPCPSCPAPEFLGGPSVFLFPPKPKDTLMISRTPEVTCVVVDVSQEDPEVQFNWYVDGVEVHNAKTKPREEQFNSTYRVVSVLTVLHQDWLNGKEYKCKVSNKGLPSSIEKTISKAKGQPREPQVYTLPPSQEEMTKNQVSLTCLVKGFYPSDIAVEWESNGQPENNYKTTPPVLDSDGSFFLYSRLTVDKSRWQEGNVFSCSVMHEALHNHYTQKSLSLSLGK

**Table 1-2 Supplementary**

**B22680402 (26.4-26.4-IgG1-LALAPG)**

MHSSALLCCLVLLTGVRA

QVQLQQSGPGLVKPSQTLSLTCAISGDSVSSNSAAWNWIRQSPSRGLEWLGRTYFRSNWYNDYAASVKSRITINQDTSKNQLSLQLNSVTPEDTAMYYCARDGAWGGSSWWPGLPHHYYSGMDVWGQGTTVTVSS

ASTKGPSVFPLAPSSKSTSGGTAALGCLVKDYFPEPVTVSWNSGALTSGVHTFPAVLQSSGLYSLSSVVTVPSSSLGTQTYICNVNHKPSNTKVDKKVEPKSCDKTHTCPPCPAPEAAGGPSVFLFPPKPKDTLMISRTPEVTCVVVDVSHEDPEVKFNWYVDGVEVHNAKTKPREEQYNSTYRVVSVLTVLHQDWLNGKEYKCKVSNKALGAPIEKTISKAKGQPREPQVYTLPPSRDELTKNQVSLTCLVKGFYPSDIAVEWESNGQPENNYKTTPPVLDSDGSFFLYSKLTVDKSRWQQGNVFSCSVMHEALHNHYTQKSLSLSPGK

**All five antibodies share the same light chain:**

>F22474201L

MHSSALLCCLVLLTGVRAEIVLTQSPATLSLSPGERATLSCRASQSVSSYLAWYQQKPGQAPRLLIYDASKRATGIPARFSGSGSGTDFSLTIRSLEPEDFAVYYCQQRSDWQGLTFGGGTKVEIKRTVAAPSVFIFPPSDEQLKSGTASVVCLLNNFYPREAKVQWKVDNALQSGNSQESVTEQDSKDSTYSLSSTLTLSKADYEKHKVYACEVTHQGLSSPVTKSFNRGEC

| **FcgRIIIa**  **(V_158_F)** | **D67**  **FV** | **D68**  **FF** | **D69**  **FV** | **D70 FV** | **D71 FV** | **D72  FV** | **D73 FV** | **D78 FV** | **D79 FV** | **D80 FV** | **D81**  **FF** | **D82 FV** | **D83 FV** | **D87 FV** | **D88 FV** |
| --- | --- | --- | --- | --- | --- | --- | --- | --- | --- | --- | --- | --- | --- | --- | --- |
| hIgG | 1.7% | 1.3% | 1.7% | 1.5% | 1.3% | 1.3% | 1.1% | 1.6% | 1.6% | 1.2% | 1.5% | 1.6% | 1.4% | 1.2% | 1.2% |
| 26.4-IgG1 LALAPG | 2.0% | 1.3% | 0.8% | 0.5% | 1.2% | 0.9% | 3.1% | 0.7% | 0.4% | 0,2% | 0,4% | 0,8% | 1.1% | 1.1% | 0.8% |
| 26.4-IgG1 | 66.1% | 53.0% | 29.4% | 30.5% | 57.9% | 79.2% | 41.5% | 25.2% | 49.3% | 65.8% | 38.9% | 84.9% | 71.9% | 54.6% | 71.1% |
| 26.4-IgG2 | 29.9% | 16.5% | 21.1% | 9.3% | 27.2% | 8.4% | 21.9% | 20.5% | 28.9% | 37.0% | 39% | 32.2% | 59.5% | 6.40% | 8.60% |
| 26.4-IgG3 | 78.8% | 60.7% | 84.3% | 89.2% | 78.1% | 90.5% | 69.1% | 72.3% | 64.0% | 71.5% | 44.8% | 91.7% | 78.6% | 87.9% | 90.7% |
| 26.4-IgG4 | 52.2% | 16.3% | 26.6% | 17.9% | 54.2% | 54.8% | 19.7% | 9.1% | 30.0% | 10.1% | 11.6% | 78.5% | 59.1% | 57.5% | 68.3% |

**Table 2 Supplementary**
