# Supplementary figures and images for: Anti‐HPA‐1a IgG3 subclass antibodies induce strong platelet phagocytosis
Source: Br J Haematol. 2026 May 14;209(1):358–62. doi: 10.1111/bjh.70533 (PMC13340540; doi:10.1111/bjh.70533)

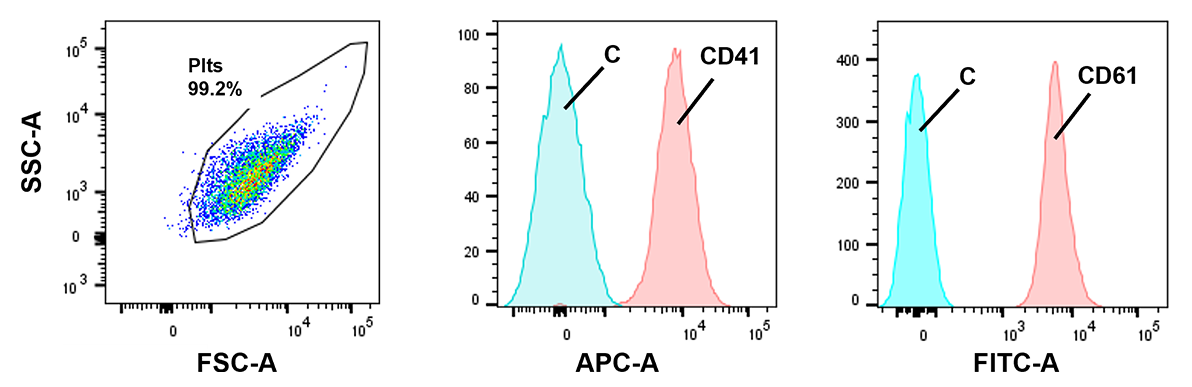

Supplement: Supplementary file 1 — Data S1. [file BJH-209-358-s001.zip › bjh70533-sup-0001-FigureS1@Figure 1 supplementary.png]

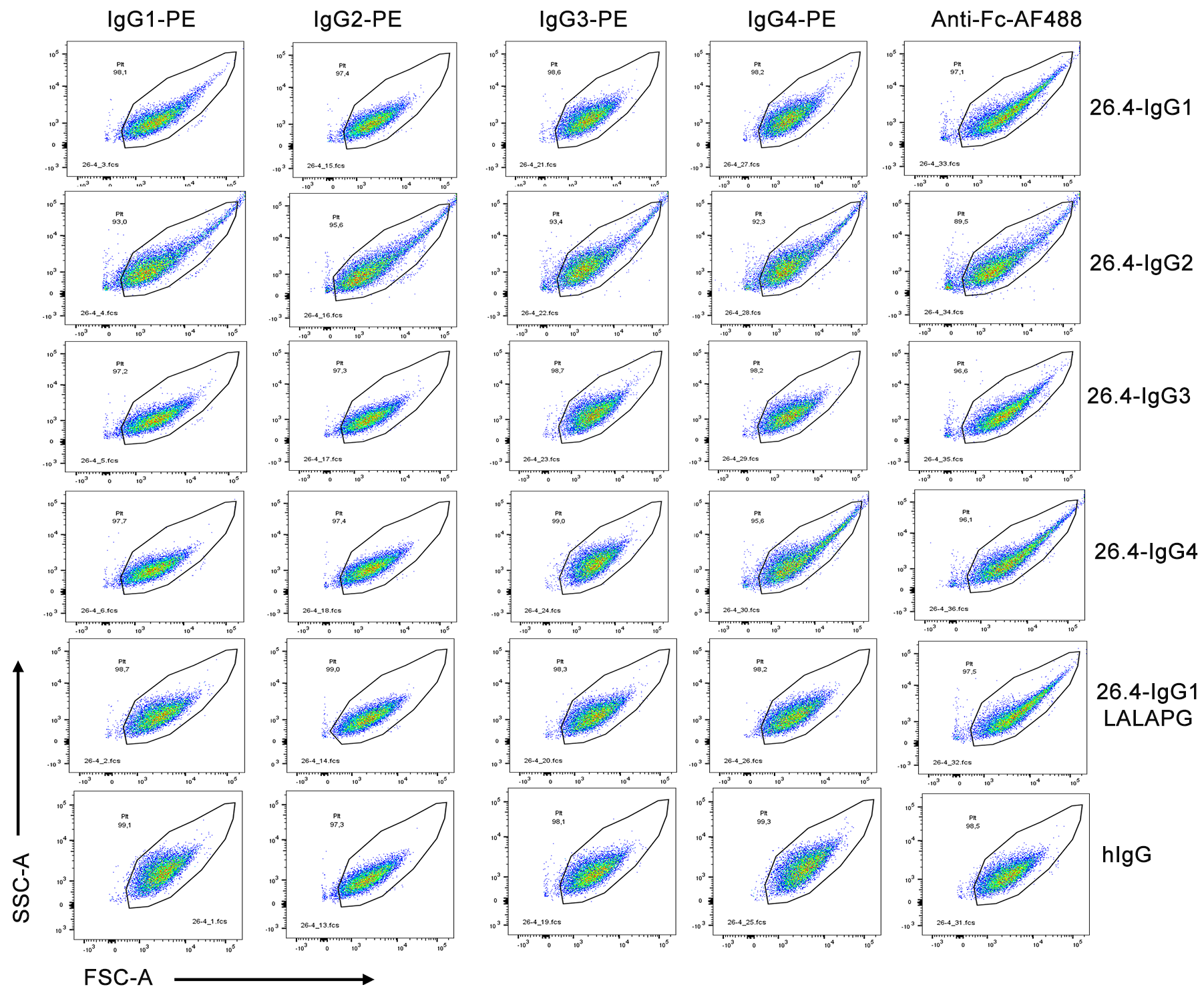

Supplement: Supplementary file 1 — Data S1. [file BJH-209-358-s001.zip › bjh70533-sup-0002-FigureS2@Figure 2 supplementary.png]
